# Evaluation of Beneficial and Adverse Effects of a Diet Supplemented with Schisandrae Fructus Seed Ethanol Extract on Lipid and Glucose Metabolism in Normal and Hypercholesterolemic/Hyperglycemic Mice

**DOI:** 10.1155/2021/8858962

**Published:** 2021-02-20

**Authors:** Xiao-Yan Wang, Xue-Lan Song, Yi Zhang, Gan Luo, Hai-Chuan Tai, Zhao-Heng Lin, Pei-Li Zhu, Nan Sun, Zhu-Sheng Chu, Zhi-Ling Yu, Si-Yuan Pan, Jin-Fa Tang, Kam-Ming Ko

**Affiliations:** ^1^The First Affiliated Hospital of Henan University of Traditional Chinese Medicine, Zhengzhou, China; ^2^School of Traditional Dai-Thai, West Yunnan University of Applied Sciences, Jinghong, China; ^3^School of Chinese Materia Medica, Beijing University of Chinese Medicine, Beijing, China; ^4^School of Chinese Medicine, Hong Kong Baptist University, Hong Kong, China; ^5^Division of Life Science, Hong Kong University of Science & Technology, Hong Kong, China

## Abstract

Schisandrae Fructus (SF), the fruit of *Schisandra chinensis* (Turcz.) Baillon, has been used for the treatment of liver injury and metabolism-related disorders in China. The objective of this study was to investigate the effects of supplementation with ethanol extract of SF seed (EtSF-S) on serum/hepatic lipid and glucose levels as well as fecal total cholesterol (TC) contents in mice fed a normal diet (ND) or high-fat/fructose diet (HFFD) containing 15% lard oil and 15% fructose. Female ICR mice (18–20 g in body weight) were fed with ND or HFFD for 3 months, and then EtSF-S was added to both chow diets at increasing concentrations of 1, 5, and 10% (w/w). Thirty days later, serum and hepatic lipids, including TC, triglyceride (TG), high-density lipoprotein (HDL), low-density lipoprotein (LDL), and glucose, were measured. Dietary supplementation with EtSF-S reduced hepatic TC (36 and 18%) and TG levels (38 and 28%) and increased serum HDL/LDL ratio (16 and 26%) in both ND- and HFFD-fed mice, respectively. Moreover, supplementation with EtSF-S elevated serum HDL (31%) in HFFD-fed mice and reduced serum LDL (27%) in ND-fed mice. EtSF-S treatment reduced fat mass (40%) in ND-fed mice and increased fecal TC contents (33%) in HFFD-fed mice. EtSF-S supplementation decreased hepatic glucose contents (29%) in both ND- and HFFD-fed mice. However, diet supplemented with EtSF-S elevated serum TG levels (up to 123%) and hepatic size (28%), but more importantly, suppressed the body weight gain (approximately 130%) in mice fed with HFFD. These findings suggested that dietary supplementation with EtSF-S as natural herbal function food may be a useful strategy for the treatment of patients with fatty liver disease or overweight without a high intake of sugar and fat.

## 1. Introduction

A high intake of sugar and fat, as in the case of Western diets, is associated with an increased incidence of obesity, hyperlipidemia, coronary heart disease (CHD), diabetes, and nonalcoholic fatty liver disease (NAFLD) [1, 2], which may result in other related diseases. For example, NAFLD, an emerging and commonly occurring clinical syndrome, includes a wide spectrum of liver diseases ranging from simple fatty liver to nonalcoholic steatohepatitis, even fibrosis, cirrhosis, and hepatocellular carcinoma [3]. In addition, patients suffering from NAFLD bear a higher risk of developing myocardial infarction and stroke [4]. Coincidentally, patients with NAFLD seem to be at a higher risk of developing type 2 diabetes mellitus than people without NAFLD [5]. Therefore, it is of great therapeutic interest and urgency to search for new drugs used for lowering lipid contents in the blood and liver. In this connection, over the last few decades, many herbal medicines have attracted much attention and research interest because of their time-tested health benefits and safety [6, 7].

Schisandrae Fructus (SF), the fruit of *Schisandra chinensis* (Turcz.) Baillon, has been used as health food for the treatment and prevention of liver injury, diabetes mellitus, and other metabolism-related disorders in China [8]. The pharmacological profile of SF and its related active compounds encompasses analgesic activity [9], neuroprotective effect [10], anxiolytic effect [11], hepatoprotective effect [12], and antioxidation [13]. Besides, it has been found that SF-derived polysaccharides reduced body weight, fatty index, and blood lipid levels in obese mice [14]. Our previous studies have demonstrated that SF extracts [15, 16] and its related compounds such as schisandrin B (Sch B) [17], bicyclol [18], and bifendate [19] reduced the hepatic lipid contents in normal and hypercholesterolemic mice. Both bicyclol and bifendate are synthetic intermediates of dibenzocyclooctene lignan for treating liver injury caused by viruses and chemicals in clinical situations.

Western-style dietary habit, which is characterized by high intakes of sugary desserts, high-sugary drinks, and high-fat foodstuff, is adopted by most of the people in developed countries and wealthy people in developing countries. According to Chinese Materia Medica as well as the Chinese herbal medicine theory, the SF seed and its pulp are sometimes used differently in the practice of Chinese medicine. In a previous study, we evaluated the effects of SF pulp on lipid and glucose metabolism in normal and hypercholesteremic animals [20]. In the present study, however, we endeavored to investigate the effects of dietary supplementation with the ethanol extract of SF seed (EtSF-S) on serum and hepatic lipid/glucose levels, fecal cholesterol contents, and fatty and hepatic mass in female mice fed with a normal diet (ND) and high-fat/fructose diet (HFFD) that mimics the Western-style diet. Also, lipid-regulating agent fenofibrate (FF), a clinically prescribed therapeutic agent for patients with hypertriglyceridemia and mixed hyperlipidemia in Western medicine, was used as a positive control for comparison with EtSF-S supplementation regarding the effects on serum/hepatic lipid parameters and glucose content.

## 2. Materials and Methods

**Figure 1:**
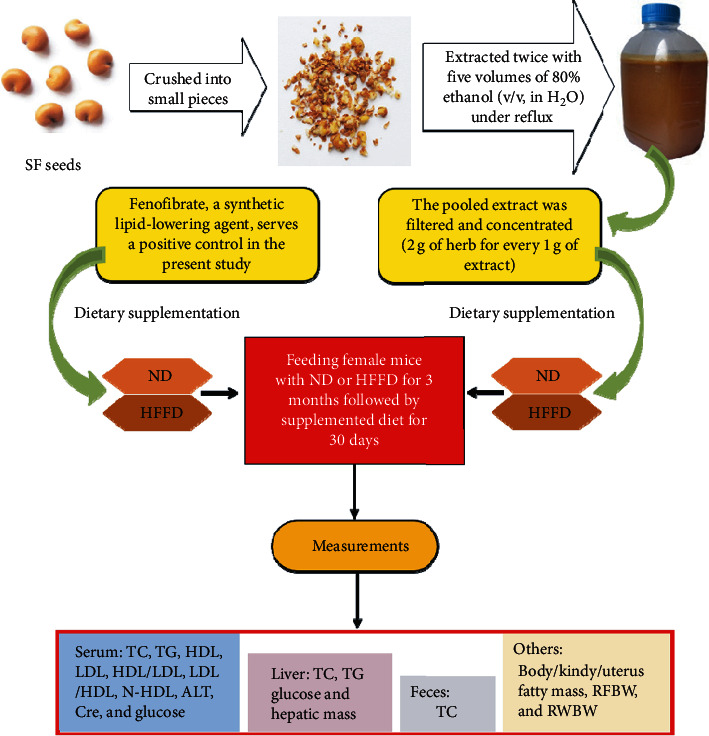
The experimental protocol of the present study.

Figure 1 shows the experimental protocol in the present study.

### 2.1. Herbal Material and Extraction

SF, the fruit of *Schisandra chinensis* (Turcz.) Baillon (batch number: 140801), was purchased from Beijing ShiZhenTang Pharma Co., Ltd. (Beijing, China) and authenticated by Professor Chun-Sheng Liu (Department of Pharmacognosy, School of Chinese Materia Medica, Beijing University of Chinese Medicine, Beijing, China) according to the guidelines of the Chinese Pharmacopoeia (2015 version). A voucher specimen (SF20140225) was kept in the Department of Pharmacology, School of Chinese Materia Medica, Beijing University of Chinese Medicine. The fruit pulp and seed were manually separated and then dried at room temperature. Dried seeds of SF were pulverized with a grinder and extracted twice (first, 1.5 h; second, 2 h) with 5 volumes of 80% ethanol (v/v, in H_2_O) under reflux after soaking for half an hour. The pooled extract was filtered by vacuum filtration with a 150 mm diameter Buchner funnel and concentrated by rotary evaporation under reduced pressure to obtain the ethanol extract of SF seed (EtSF-S) at a yield of 50% (w/w) and then stored at 4°C until use (up to two months).

### 2.2. Chemical Characterization of EtSF-S

**Figure 2:**
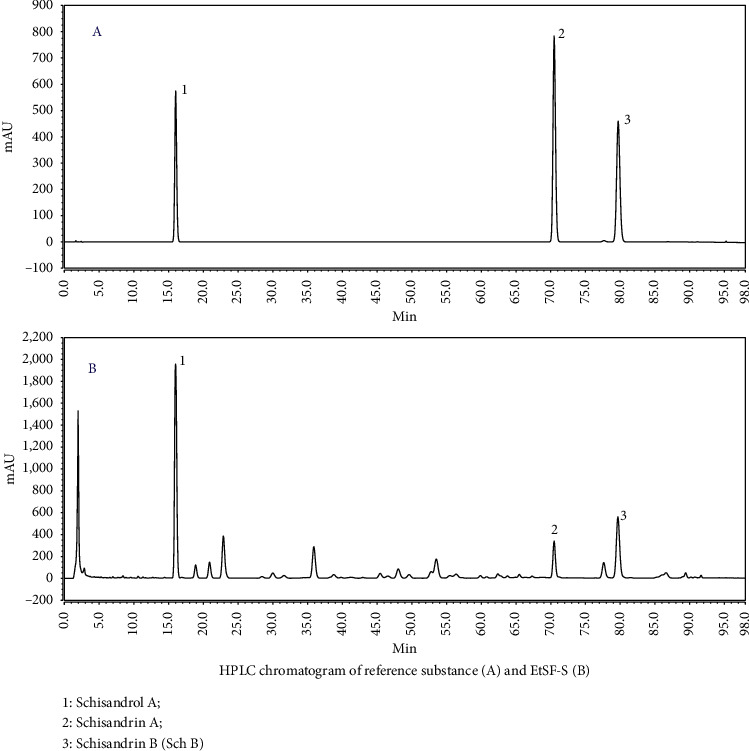
HPLC chromatogram of reference substance (a) and EtSF-S (b). (1) Schisandrol A; (2) schisandrin A; (3) schisandrin B (Sch B).

Up till now, the lignan ingredients presented in SF seed, such as schisandrol A, schisandrin A (Sch A), and Sch B, are considered the most abundant and very biologically important compounds. Therefore, the contents of these lignans in EtSF-S were measured as follows: EtSF-S (2.0547 g) was taken to a 10 mL volumetric flask after a precise weighing, dissolved by 80% (v/v) ethyl alcohol, and then diluted with 80% (v/v) ethyl alcohol to a final volume of 10 mL. Then, 1 mL of the solution was mixed with 24 mL of methyl alcohol and filtered through a Millipore membrane filter with an average pore diameter of 0.22 *μ*m, and 10 *μ*L filtrate was injected into the HPLC system for analysis. The contents of schisandrol A, Sch A, and Sch B were quantitated by the reverse phase HPLC. An Ultimate 3000 UHPLC system (Thermo Fisher Scientific, Waltham, Massachusetts, USA) with a vacuum degasser, a quaternary pump, an autosampler, a thermostatic column compartment, and a diode array detector was used. The separation was performed on Agilent SB C_18_ column (4.6 mm × 250 mm, 5 *μ*m particle size) at 35°C. The gradient elution profile was performed as follows: 0–30 min, 43% A; 30–32 min, 43–46% A; 32–53 min, 46–50% A; 53–64 min, 50–62% A; 64–83 min, 62% A; 83–93 min, 62–90% A; 93–98 min, 90–100% A. The flow rate was 1.0 mL/min, with the column temperature maintained at 35°C. Reequilibration duration was 10 min between individual runs. The results indicated that every gram of EtSF-S contained schisandrol A (89.6 mg), Sch A (17.8 mg), and Sch B (38.8 mg) as shown in Figure 2.

### 2.3. Chemicals and Regents

FF (certificate number 20667) was bought from Beijing Yongkang Medical Ltd. (Beijing, China). Assay kits for total cholesterol (TC, certificate no.131601), triacylglycerol (TG, certificate no. 136281), and glucose (certificate no. 133071) were obtained from Zhongsheng Beikong Biotechnology and Science Inc. (Beijing, China). Assay kits for high-density lipoprotein (HDL, certificate no. 303142B) and low-density lipoprotein (LDL, certificate no. 302271A) were purchased from Beijing Leadman Biochemistry Co., Inc. (Beijing, China).

### 2.4. Animals

Female ICR mice (18–20 g) were purchased from Vital River Lab Animal Co. Ltd. (Beijing, China) and maintained in the animal facility at the Beijing University of Chinese Medicine. Animals were housed with a 12 h light/dark cycle at 20–22°C, with a relative humidity of 50–55%. They were allowed for free access to water and food. Blood samples and liver, fat, and kidney tissue samples were obtained from ether-anesthetized animals which had been fasted for 6 h (from 06 : 00 to 12 : 00), and they were subjected to biochemical analyses. All experimental protocols were approved by the University Animal Care Committee for Animal Research in Beijing University of Chinese Medicine.

### 2.5. Chow Diet

The regular chow (normal diet, ND), which was purchased from Vital River Lab Animal Co. Ltd. (Beijing, China), was composed of 20% protein, 4.5% fat, 54% carbohydrates in mass ratio or 23.8% protein, 12.0% fat, and 64.2% carbohydrates in energy ratio (total energy is 3.37 kcal/g). HFFD was constituted of 70% ND, 15% fat (lard oil), and 15% fructose, which was freshly prepared and stored at room temperature.

### 2.6. Experimental Protocol

First experimental protocol: this study aimed to investigate the effects of EtSF-S or FF supplementation on lipid and glucose metabolism in mice fed with a standard diet. Here, mice were divided into 3 groups of 10 animals in each: (1) mice fed with ND as the control group; (2) mice fed with ND supplemented with 10% (w/w) EtSF-S; (3) mice fed with ND supplemented with 0.03% (w/w) FF as the positive control group. The body weight and the amounts of food/water intake were measured every three days. Thirty days after supplementation with EtSF-S or FF, animals were sacrificed and blood/tissue samples were obtained for biochemical analyses or weight measurement.

Second experimental protocol: this study aimed to investigate the effect of EtSF-S or FF supplementation on lipid and glucose metabolism in mice fed with HFFD. The mice were first fed with either HFFD or ND for 3 months. After that, they were randomly divided into six groups with 10 mice in each: (1) mice fed with ND as the control (normal) group; (2) mice fed unceasingly with HFFD as an animal model of hypercholesterolemia and hyperglycemia; (3), (4), and (5) mice fed with HFFD supplemented with 1, 5, and 10% EtSF-S, respectively; (6) mice fed with HFFD supplemented with 0.03% FF as the positive control group. Thirty days following the supplementation with EtSF-S or FF, relevant parameters of blood and tissue samples were determined.

### 2.7. Biochemical Analyses

Serum samples were prepared by centrifuging the whole blood obtained from the orbital vein for 8 min at 2000 × *g* and 4°C and then stored at −20°C until the use for biochemical analyses within 5 days. Liver tissue samples were homogenized in 9 volumes of 0.9% (w/v) NaCl solution by two 10 s bursts of a tissue disintegrator at 13,500 rpm. Then, the homogenates were divided into three portions. One portion was centrifuged at 2000 × *g* for 15 min at 4°C to obtain the supernatant. The two other portions were stored at 4°C and −20°C, respectively, for 24 h. They were then centrifuged for 15 min at 2000 × g at 4°C to obtain the supernatant for the measurement of glucose levels. Forty *μ*L of the hepatic supernatant and 10 *μ*L serum were used to determine TG and TC levels with GPO-PAP (glycerol-3-phosphate oxidase and phenol + aminophenazone) and COD-PAP (cholesterol oxidase phenol 4-aminoantipyrine peroxidase) methods according to the manufacturer's manual, respectively. Ten *μ*L serum and hepatic supernatant were used to determine glucose levels with the GOD-POD method. Serum HDL and LDL levels, as well as alanine aminotransferase (ALT) activity and creatinine level, were determined by using automatic Biochemistry Analyzer (Beckman Coulter Synchron CX4 PRO. Brea, CA, USA). HDL/LDL, LDL/HDL, and non-HDL (N-HDL) values were estimated by calculating the ratio of HDL to LDL and LDL to HDL, and the difference in values between TC and HDL as well, respectively.

### 2.8. Measurement of Fecal TC

Feces of each mouse were dried at room temperature and ground into powder. About 30 mg of fecal samples was extracted with 0.5 mL chloroform/methanol (1/1, v/v) mixture for 12 h and centrifuged at 2000 × *g* for 5 min. Then, 30 *μ*L of the supernatant was used to measure the TC concentration (*μ*mol/g feces), according to the COD-PAP method.

### 2.9. Measurement of Liver, Kidney, and Uterus Fat Mass/Index

Weights of the liver, kidney, uterine fat, and the whole body were measured. The organ/fat index was estimated from the ratio of organ or fat weight to body weight × 100, i.e., (organ or fat weight/body weight) × 100, or the ratio of organ or fat weight to body weight minus the hepatic weight, i.e., (organ or fat weight/body weight ‒ hepatic weight) × 100.

### 2.10. Measurement of RFBW and RWBW

The ratio of food intake to body weight (RFBW) was calculated by using the formula as follows: total food (g) intake divided total body weight (g) for an experimental period of 30 days. The ratio of water intake to body weight (RWBW) was calculated by using the following formula: total water (mL) intake divided total body weight (g) for an experimental period of 30 days.

### 2.11. Statistical Analysis

All data were expressed as the mean ± SD, and they were analyzed by either one-way ANOVA followed by Dunnett's multiple comparison test or Student's *t*-test to detect intergroup differences, using SPSS statistical analysis program. *p* value <0.05 was considered statistically significant.

## 3. Results

### 3.1. Body Mass, RWBW, RFBW, and Drug Intake

The amount of food intake can affect lipid and glucose levels in the blood and liver, as well as body mass. In this study, the estimation of food and water intake and the ratio of food intake to body weight (RFBW) and water intake to body weight (RWBW) were performed. During the experimental period of supplementation, the mean body weight gain was decreased by 0.91 g in ND-fed mice, but it was increased by 0.77 g in HFFD-fed mice (*p* < 0.01). EtSF-S supplementation produced no detectable effect on body weight gain in both ND- and HFFD-fed mice. However, the body weight gain was increased by 109% in mice fed with HFFD supplemented with FF (*p* < 0.05). Daily intakes of water and food were estimated to be 131–187 mL/kg/day and 76–134 g/kg/day, respectively. Dietary consumption of EtSF-S increased the amounts of water and food intake in a dose-dependent manner in the mice fed with HFFD. RFBW and RWBW were significantly increased (up to 56 and 43%, respectively) in the mice fed with HFFD supplemented with EtSF-S, when compared with the mice fed with HFFD alone. In this study, it was found that HFFD-fed mice showed decreases in food and water intake, as well as the values of RFBW and RWBW (approximately 21 and 26%, respectively), when compared with the ND-fed mice. The amounts of daily intake of EtSF-S and FF were also estimated based on the amount of ingested diet (g/kg/d) and the drug concentration in the diet (Table 1).

### 3.2. Serum, Hepatic, and Fecal Lipids

**Figure 3:**
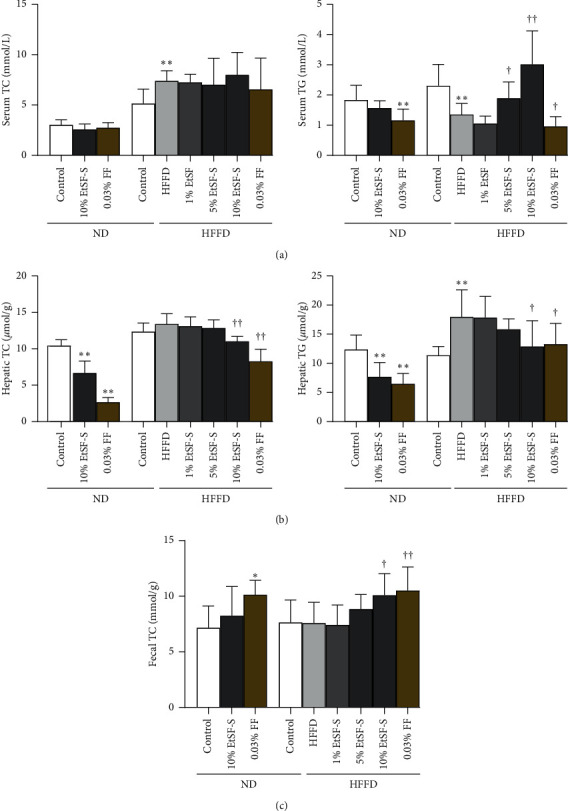
Effect of dietary supplementation with EtSF-S or FF on serum, hepatic, and fecal lipids in ND- and HFFD-fed mice. Mice were fed with normal diet (ND) or high fructose/fat diet (HFFD) for 3 months. Then, animals were fed with ND or HFFD without and with supplementation with the ethanol extract of Schisandrae Fructus (SF) seed (EtSF-S) or fenofibrate (FF). After 30 days of experiment, serum and hepatic total cholesterol (TC) and triacylglycerol (TG) levels including fecal TC contents were measured. The concentrations of EtSF-S in chow diet were estimated on the basis of crude herbal material (SF seed). Values given are the mean ± SD, with *N* = 10. ^*∗*^*p* < 0.05, ^*∗∗*^*p* < 0.01 vs. ND alone; ^†^*p* < 0.05, ^††^*p* < 0.01 vs. HFFD alone.

Lipid metabolic disorder is the root cause of many modern diseases. In the clinical situation, SF is usually prescribed for improving human health status, especially in individuals with weak body function, including those suffering from dyslipidemia. In the present study, serum, liver, and fecal lipid levels were measured after dietary supplementation with EtSF-S or FF. Although feeding mice with HFFD caused the elevation of serum TC level by 44% (*p* < 0.01), supplementation with EtSF-S or FF for 30 days did not alter the levels of serum TC in ND- and HFFD-fed mice (Figure 3(a)). HFFD intake reduced serum TG level by 41% (*p* < 0.01), when compared with the ND-fed mice. Supplementation of EtSF-S in HFFD-fed mice elevated the level of serum TG in a dose-dependent manner (up to 123%, *p* < 0.01). FF reduced serum TG levels by 37 (*p* < 0.01) and 29% (*p* < 0.05) in ND- and HFFD-fed mice, respectively (Figure 3(a)). Mice fed ND supplemented with 10% EtSF-S for 30 days showed a significant decrease in hepatic TC (36%, *p* < 0.01) and TG (38%, *p* < 0.01) levels. Feeding mice with HFFD markedly increased hepatic TG contents (up to 52%, *p* < 0.01), when compared with the ND-fed mice. HFFD supplemented with 10% EtSF-S significantly decreased hepatic TC and TG contents (by 18 and 28%, *p* < 0.01 and *p* < 0.05, respectively), when compared with the HFFD-fed alone (Figures 3(b)). EtSF-S supplementation largely increased fecal TC content (up to 33%, *p* < 0.05) in a dose-dependent manner in HFFD-fed, but not ND-fed, mice. Feeding mice with FF also markedly increased fecal TC content in both ND- and HFFD-fed mice (by 26 and 38%, respectively) (Figure 3(c)).

### 3.3. Serum Lipoproteins

It is well known that blood and liver lipid levels are influenced by lipoproteins. In the present study, serum HDL and LDL levels as well as other related parameters were also determined or estimated. Results (Table 2) showed that supplementation with EtSF-S for 30 days lowered serum LDL level (27%, *p* < 0.01), LDL/HDL ratio (14%, *p* < 0.05), and N-HDL levels (35%, *p* < 0.01) but elevated serum HDL/LDL ratio (16%, *p* < 0.05) in mice fed with ND. Feeding mice with HFFD elevated serum HDL (43%, *p* < 0.01), LDL (85%, *p* < 0.01), LDL/HDL ratio (32%, *p* < 0.01), and N-HDL (34%, *p* < 0.01) but lowered HDL/LDL ratio by 25% (*p* < 0.01), when compared with the ND-fed mice. Serum levels of HDL and HDL/LDL ratio were increased (up to 31 and 24%, *p* < 0.01, *p* < 0.05, respectively) in a dose-dependent manner, but LDL/HDL ratio was reduced by 24% (*p* < 0.01) in mice fed with HFFD supplemented with EtSF-S, when compared with the non-EtSF-S supplemented HFFD-fed control. FF supplement decreased serum LDL, N-HDL, and LDL/HDL ratio (by 29, 36, and 29%; *p* < 0.051, *p* < 0.05, and *p* < 0.01; respectively) but increased the HDL/LDL ratio by 39% (*p* < 0.01) in ND-fed mice. HFFD supplemented with FF elevated serum LDL levels and LDL/HDL ratio by 27 and 33% (*p* < 0.05), respectively, but serum HDL/LDL ratio lowered by 27% (*p* < 0.05), when compared with the non-FF supplemented HFFD.

### 3.4. Serum and Hepatic Glucose

It is well established that lipid/fat metabolism is closely related to glucose metabolism in the body. Here, we measured the alteration in serum and hepatic glucose levels in normal and hypercholesterolemic/hyperglycemic mice after EtSF-S or FF treatment. Supplementation with EtSF-S for 30 days did not alter the levels of serum glucose; however, FF supplementation increased serum glucose levels by 15% (*p* < 0.01) in ND-fed mice. Feeding mice with HFFD elevated serum glucose levels by 19% (*p* < 0.05). EtSF-S supplementation caused an elevation in serum glucose levels (up to 18%, *p* < 0.05) in a dose-dependent manner in HFFD-fed mice. FF supplement did not alter the levels of serum glucose in HFFD-fed mice (Table 3A).

Table 3B shows the effect of EtSF-S and FF supplementation on hepatic glucose contents, including liver tissue samples which had been stored for 24 h at 4°C and −20°C. In freshly harvested liver samples, it was found that EtSF-S and FF supplementations reduced hepatic glucose level by 16 and 44% (*p* < 0.05 an *p* < 0.01), respectively, in ND-fed mice. Feeding mice with HFFD reduced hepatic glucose content by 11% (*p* < 0.01), when compared with the ND-fed mice. EtSF-S supplementation lowered the hepatic glucose content (by approximately 29%, *p* < 0.01) in a dose-dependent manner in HFFD-fed mice. HFFD supplemented with FF reduced hepatic glucose content by 29% (*p* < 0.01), when compared with HFFD without supplementation. After storing for 24 h at −20°C and 4°C, glucose level contents of all liver tissue samples were elevated (up to 39 and 162%, *p* < 0.01, respectively) in a temperature-dependent manner, when compared with the unstored (i.e., fresh) samples. Alterations of glucose contents in the stored samples obtained from EtSF-S and FF supplemented mice were similar to the unstored samples.

### 3.5. Hepatic, Renal, and Uterine Fat Mass

The liver is a very peculiar organ involved in lipid and glucose metabolism. Here, we investigated the effect of EtSF-S and FF supplementation on hepatic weight and function. While feeding mice with 10% EtSF-S did not affect hepatic weight in the mice fed with ND, EtSF-S supplementation at doses of 5 and 10% increased hepatic weight/index by 15/21 and 28/38% (*p* < 0.05 and *p* < 0.01) in HFFD-fed mice, respectively. ND and HFFD supplemented with FF significantly increased hepatic mass/index by 44/44 and 29/28% (*p* < 0.01), respectively (Table 4A). Because enlarged livers were found after EtSF-S or FF supplementation, weights of other organs and uterine fat were also measured. While feeding mice with EtSF-S and FF lowered fat mass/index (approximately 40/38 and 44/43% (*p* < 0.01), respectively) in mice fed with ND, they did not alter the fat mass/index in mice fed with HFFD (Table 4B). EtSF-S supplementation at 10% decreased the renal index by 11% (*p* < 0.05) in HFFD-fed mice. FF supplementation increased renal index by 11% (*p* < 0.05) in ND-fed mice and 26% (*p* < 0.05) in HFFD-fed mice (Table 4C). However, both EtSF-S and FF supplementation did not affect the heart, lung, spleen, and uterus masses (data not shown). Since EtSF-S and FF supplementations altered the weights of the liver and kidney, serum ALT activity (an indicator of liver function) and creatinine (an indicator of renal function) levels were determined. Results showed that there were no significant differences in serum ALT activity and creatinine levels between the mice fed with EtSF-S or FF supplementation and the mice fed with nonsupplemented ND and HFFD (data not shown).

## 4. Discussion

In the present study, feeding mice with HFFD for 4 months, including 3 months for establishing hypercholesterolemic/hyperglycemic animal model and one month of drug treatment, caused elevations in serum TC, HDL, LDL, N-HDL, and glucose levels, as well as hepatic TG contents. However, serum TG and hepatic glucose levels were decreased in HFFD-fed mice. Studies have shown that the administration of HFFD caused a significant increase in blood TG levels in experimental animals [21, 22]. In contrast, our results indicated that serum TG levels were decreased while hepatic TG content was increased after feeding mice with HFFD for 4 months. The species and gender differences of experimental animals, as well as the duration and amount of fat/fructose intake, may contribute to the discrepant observation. While the body weight gain in HFFD-fed mice was larger than that in ND-fed mice during the period of EtSF-S or FF supplementation, only around 20% of mice prefed with HFFD developed severe overweight and visceral adiposity. The results suggested that a differential susceptibility of mice to HFFD-induced obesity exists. In clinical situations, it is well known that dietary high sugar/fat consumption may lead to overweight/obesity in some but not all individuals. This observation might be related to the differences among subjects in carbohydrate absorption and metabolism, which are regulated by individual gut microbes and genetic characteristic [23, 24].

In the present study, we found that HFFD could raise body weight gain and feed conversion ratio, i.e., a lower value of RFBW and RWBW observed in HFFD-fed mice, which might result from a higher percentage of fat and sugar with higher calories/energies in the diet. However, EtSF-S supplementation increased the food and water intake in HFFD-fed mice, but it did not increase their body weight. Therefore, higher values of RFBW and RWBW were observed in the mice fed with HFFD supplemented with EtSF-S. This observation suggested that some ingested food/energy might not be converted into body weight and adipose tissue mass by consuming EtSF-S supplemented HFFD. SF seed may, therefore, be utilized as a prophylactic or therapeutic agent in overweight individuals or people who are eager to maintain a healthy body weight. However, this postulation needs to be confirmed by further studies. Both EtSF-S and FF supplementation reduced the adipose mass in ND-fed mice, but they did not cause the same in HFFD-fed mice. It suggested that HFFD might inhibit the EtSF-S- and FF-induced fat loss. Conceivably, an individual aims to lose weight by therapeutic intervention should avoid consuming the foodstuff containing high levels of fat and sugar. The observation suggests that EtSF-S may be potentially used as a slimming product for those who do not eat foodstuff with too much fat and sugar.

It is widely accepted that LDL is linked to the development of atherosclerosis, making it a “bad” cholesterol [25]. Currently, it is believed that N-HDL, which is a more reliable marker than LDL in assessing cardiovascular risk, can be a therapeutic target in patients with diabetic dyslipidemia [26, 27]. Also, abnormal changes in LDL/HDL or HDL/LDL ratio, which is commonly used as a parameter for estimating the risk of atherosclerotic cardiovascular disease, is associated with obesity, diabetes, NAFLD, and metabolic syndrome [28]. In this study, the intake of HFFD was found to increase the LDL/HDL ratio and decrease the HDL/LDL ratio, which is consistent with the clinical manifestation of NAFLD. While dietary EtSF-S did not affect serum levels of TC and TG, it reduced serum levels of LDL, N-HDL, and LDL/HDL ratio and elevated HDL/LDL ratio in ND-fed mice. EtSF-S supplementation increased serum HDL and HDL/LDL ratio but lowered serum LDL/HDL ratio in HFFD-fed mice. EtSF-S supplementation-associated higher blood levels of HDL cholesterol and HDL/LDL ratio may also contribute to a wide range of biological activities, including antioxidation, anti-inflammation, proendothelial function, antithrombosis, modulation of immune function, and vasorelaxation, and all of which make HDL a “good” cholesterol [29]. Moreover, EtSF-S supplementation reduced hepatic TG and TC contents in both ND- and HFFD-fed mice. Besides, fecal TC contents were found to increase in mice supplemented with EtSF-S, suggesting that the increase in the total amount of TC excretion via feces might also partly contribute to the hepatic lipid-lowering effect of EtSF-S. These findings indicated the beneficial effects of EtSF-S on lipid metabolism, which may be used, possibly as a food additive or functional food for treating and/or preventing hyperlipemia-related diseases, particularly fatty liver disease and obesity.

It is well established that lipid/fat metabolism is closely related to glucose metabolism in the body. The metabolic disorders resulting from a high sugar/fat diet constitute the pathological basis of modern epidemics such as hyperlipidemia, hyperglycemia, NAFLD, and obesity. In the present study, EtSF-S supplementation was found to lower hepatic glucose content. However, it elevated serum glucose levels in HFFD-fed mice fed mice but not in ND-fed mice. This observation indicated that the effect of EtSF-S on glucose metabolism would be dependent on the basal metabolic status of the body or the nature of dietary components. In clinical situations, high-carbohydrate (sugar) diet may result in hypertriglyceridemia, implicating that serum TG levels are at least related to serum glucose concentrations/metabolism. Although EtSF-S supplementation showed an increase in serum TG, but not TC, and glucose levels in HFFD-fed mice, it lowered hepatic TG contents in both ND- and HFFD-fed mice which was associated with decreases in hepatic glucose concentrations. The complexity of EtSF-S-induced alteration in TG and glucose metabolism needs further investigations. The reduction of hepatic TC levels in mice treated with EtSF-S may be related to the inhibition of lipogenesis and the increase in hepatic lipid excretion via the intestinal tract. Our results also showed that glucose concentrations in supernatants of hepatic homogenates, which had been stored at 4°C for 24 h, were much higher than those samples stored at −20°C for 24 h. The high levels of glucose in the liver tissue samples stored at 4°C are likely formed by the degradation of liver glycogen. Unfortunately, in the present study, glycogen contents of the liver were not measured. Besides, the EtSF-S supplementation-associated low hepatic glucose level might be related to the fact that EtSF-S could increase the glucose disposal rate and hepatic insulin sensitivity through activating peroxisome proliferator-activated receptor-*γ* [30].

In the current study, EtSF-S and FF supplementation increased hepatic (both EtSF-S and FF) and renal indices (FF), but they did not elevate serum ALT activity and creatinine level, suggesting that EtSF-S and FF induced increases in liver and kidney weights are not pathologically relevant at the adopted dosage (at 10% EtSF-S and 0.03% FF supplementation). In our previous study, however, FF (at 0.05% and 0.1% supplementation) increased serum ALT activity by 78% (31) and 6.5-fold (16) in hyperlipemic mice, respectively, which were associated with body weight loss and/or hepatomegaly. In the present study, the adopted dosage of FF (0.03% supplementation) was about 0.03 g/kg/d, which was 10-fold higher than the human dosage. EtSF-S 1% (0.75 g/kg/d), 5% (5.61 g/kg/d), and 10% (13.36 g/kg/d) supplementation for mice were about 7-, 56-, and 100-fold higher than the human dosage based on the clinically prescribed dose (10 g) of SF, respectively. Since medicinal herbs usually exhibit a large safety margin, the dosage used in patients may vary from a few grams to dozens of grams. Also, the highest dosage (13.36 g/kg/d) of EtSF-S administered in the present study only amounted to approximately 1/3 of its acute oral LD_50_ value (35.10 g/kg with a 95% confidence limit of 31.97–39.49 g/kg, data not shown) based on the crude herb.

Although EtSF-S is not a commonly prescribed lipid-lowering agent, it produces effects similar to FF on lipid and glucose in the ND-fed mice. Moreover, a high dosage (100-fold higher human dosage) of EtSF-S did not cause liver injury, but FF at a 30-fold higher human dosage was found to increase serum ALT activity. This means that EtSF-S has some advantages over FF for treating metabolic disorders relating to sugar and fat, particularly obesity/overweight and fatty liver disease. Due to hypertriglyceridemia and hypotriglyceridemia caused by EtSF-S and FF supplementation, respectively, in HFFD-fed mice, the combined application of EtSF-S and FF may be a good selection for some individuals with a high level of serum TG. It is well known that the generation of bioenergy is a crucial function of glucose and fat (i.e., TG) metabolism in the body. Therefore, the mechanism underlying the action of EtSF-S on fat and glucose levels, which may involve cellular energy metabolism, remains to be investigated.

## 5. Conclusion

**Figure 4:**
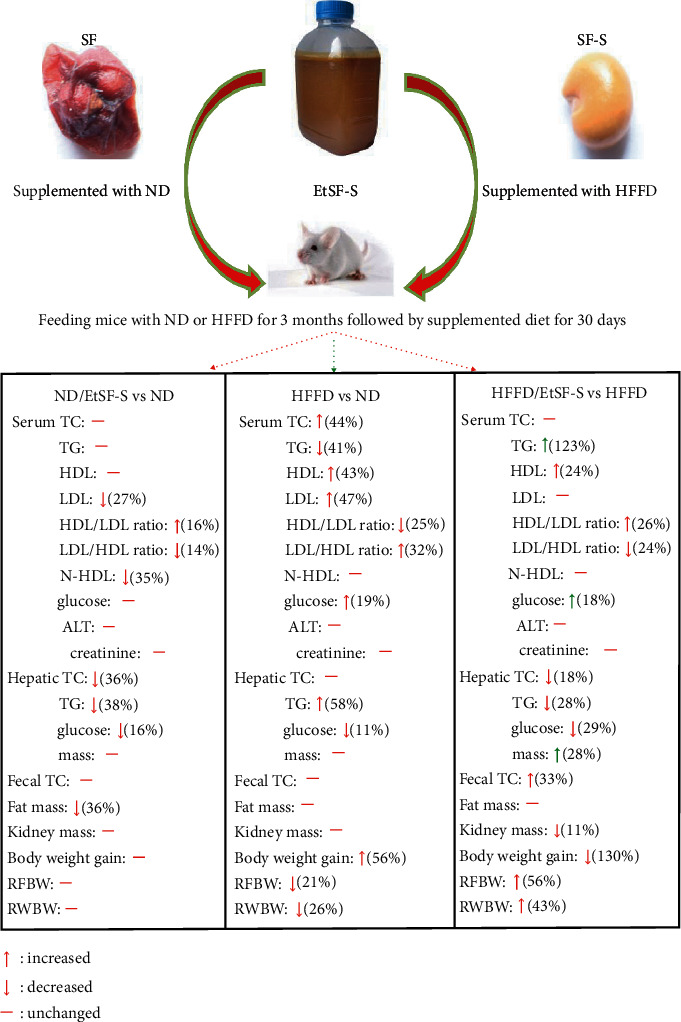
The summary of the present study.

The current study demonstrated that dietary supplementation with EtSF-S lowered the visceral fat mass in ND-fed mice as well as hepatic lipid and glucose contents in both ND- and HFFD-fed mice. EtSF-S supplementation increased serum HDL level and HDL/LDL ratio but decreased serum LDL/HDL ratio in hypercholesterolemic/hyperglycemic mice caused by HFFD feeding. The results suggest that the supplementation with EtSF-S, as a food additive or functional food, may offer a promising prospect for protecting against NAFLD, obesity, or lipid disorders (Figure 4). Given that results obtained from the present study also implicate the potential therapeutic application of SF seed on metabolic disorders of lipid/glucose, future studies are warranted to elucidate the biochemical mechanism underlying this mode of action.

## Figures and Tables

**Table 1:** Effects of dietary supplementation with EtSF-S or FF on food/RFBW, water/RWBW, and drug intake in ND- and HFFD-fed mice.

Groups	Dose (%, w/w)	Body mass (g)	Body weight gain (g)	Food		Water		Drug intake (g/kg/d)
				Intake (g/kg/d)	RFBW	Intake (mL/kg/d)	RWBW	
ND-fed mice								
ND	—	29.68 ± 1.95	‒1.09 ± 0.98	101	3.03	148	4.44	—
ND/EtSF-S	10	30.75 ± 1.95	‒0.47 ± 0.76	96	2.88	163	4.89	8.63
ND/FF	0.03	29.84 ± 1.55	‒1.59 ± 0.49	103	3.09	174	5.22	0.03

HFFD-fed mice								
ND	—	30.14 ± 4.04	‒0.91 ± 1.19	109	3.27	176	5.28	—
HFFD	—	30.47 ± 1.72	0.77 ± 0.92^*∗∗*^	86	2.58	131	3.93	—
HFFD/EtSF-S	1	30.21 ± 2.78	0.87 ± 0.96	76	2.28	138	4.14	0.75
	5	29.93 ± 2.69	0.30 ± 0.89	76	2.28	167	5.01	3.81
	10	29.39 ± 1.56	‒0.23 ± 1.44	134	4.02	187	5.61	13.36
HFFD/FF	0.03	30.73 ± 2.90	1.61 ± 0.84^†^	95	2.85	134	4.02	0.03

Mice were fed with normal diet (ND) or high fructose/fat diet (HFFD) for 3 months. Then animals were fed with ND or HFFD without and with supplementation with the ethanol extract of Schisandrae Fructus (SF) seed (EtSF-S) or fenofibrate (FF). During the experimental period of supplementation, body weight gain and the mean values of food, water, and drug intake were measured. The dosages of drugs (g/kg/d) were determined with respect to the amount of ingested diet (g/kg/d) and drug concentration in the diet. The intake amount of diet and water was recorded per cage (group). The dose of EtSF-S intake was estimated with reference to the crude herbal material (SF seed). The ratio of food and water intake to body weight (RFBW and RWBW, respectively) was calculated by using the formula as follows: total food (g) and water (mL) intake divided total body weight (g) for an experimental perimental period of 30 days. Values given are the mean ± SD, with *N* = 10. ^*∗∗*^*p* < 0.01 vs. ND alone; ^†^*p* < 0.05 vs. HFFD alone.

**Table 2:** Effect of dietary supplementation with EtSF-S or FF on serum lipoproteins in ND- and HFFD-fed mice.

Groups	Dose (%, w/w)	HDL (mmol/L)	LDL (mmol/L)	HDL/LDL	LDL/HDL	N-HDL (mmol/L)
ND-fed mice						
ND	—	2.10 ± 0.31	0.45 ± 0.10	4.81 ± 0.64	0.21 ± 0.03	0.93 ± 0.21
ND/EtSF-S	10	1.83 ± 0.40	0.33 ± 0.06 ^*∗∗*^	5.60 ± 0.89 ^*∗*^	0.18 ± 0.03 ^*∗*^	0.60 ± 0.16 ^*∗∗*^
ND/FF	0.03	1.92 ± 0.27	0.29 ± 0.04 ^*∗∗*^	6.68 ± 1.07 ^*∗∗*^	0.15 ± 0.02 ^*∗∗*^	0.66 ± 0.23 ^*∗*^

HFFD-fed mice						
ND	—	2.74 ± 0.61	0.51 ± 0.13	5.68 ± 1.16	0.19 ± 0.05	2.26 ± 0.92
HFFD	—	3.93 ± 0.55 ^*∗∗*^	0.97 ± 0.28 ^*∗∗*^	4.25 ± 0.88 ^*∗∗*^	0.25 ± 0.05 ^*∗∗*^	3.41 ± 0.74 ^*∗∗*^
HFFD/EtSF-S	1	4.07 ± 0.51	0.78 ± 0.21	5.07 ± 0.66†	0.19 ± 0.04^††^	3.10 ± 0.71
	5	4.56 ± 0.81	0.83 ± 0.19	5.36 ± 1.05^†^	0.19 ± 0.04^††^	3.49 ± 1.52
	10	5.15 ± 0.80^††^	1.04 ± 0.17	5.29 ± 0.74^†^	0.20 ± 0.04^†^	3.84 ± 0.67
HFFD/FF	0.03	4.30 ± 0.56	1.29 ± 0.21^†^	3.14 ± 0.88^†^	0.34 ± 0.09^†^	4.29 ± 1.54

Mice were fed with normal diet (ND) or high fructose/fat diet (HFFD) for 3 months. Then, animals were fed with ND or HFFD without and with supplementation with the ethanol extract of Schisandrae Fructus (SF) seed (EtSF-S) or fenofibrate (FF). After 30 days of experiment, serum high-density lipoprotein (HDL) and low-density lipoprotein (LDL) levels were measured. In addition, serum HDL/LDL ratio, LDL/HDL ratio, and non-HDL (N-HDL, i.e., TC minus HDL) levels were also determined. The concentrations of EtSF-S in chow diet were estimated on the basis of crude herbal material (SF seed). Values given are the mean ± SD, with *N* = 10. ^*∗*^*p* < 0.05, ^*∗∗*^*p* < 0.01 vs. ND alone; ^†^*p* < 0.05, ^††^*p* < 0.01 vs. HFFD alone.

**Table 3:** Effect of dietary supplementation with EtSF-S or FF on serum and hepatic glucose in ND- and HFFD-fed mice.

Groups	Dose (%, w/w)	A: serum glucose (mmol/L)	B : hepatic glucose (*µ*mol/g)		
			Fresh samples	Samples were stored for 24 h	
				At −20°C	At 4°C
ND-fed mice	–				
ND	10	5.32 ± 0.59	147.27 ± 11.16	170.80 ± 26.23	341.64 ± 51.08^##^
ND/EtSF-S	0.03	5.18 ± 0.65	123.12 ± 24.21 ^*∗*^	155.80 ± 17.43	357.66 ± 48.37^##^
ND/FF		6.14 ± 0.50 ^*∗∗*^	82.22 ± 9.40 ^*∗∗*^	114.46 ± 7.60 ^*∗∗*^	215.15 ± 43.53^*∗∗*##^

HFFD-fed mice	–				
ND		4.70 ± 0.80	160.42 ± 24.30	186.91 ± 21.70	260.22 ± 21.91^##^
HFFD		5.59 ± 1.07 ^*∗*^	142.28 ± 8.76 ^*∗*^	162.64 ± 13.66 ^*∗*^	251.56 ± 17.22^##^
HFFD/EtSF-S	1	5.33 ± 0.82	147.77 ± 17.6	170.71 ± 19.95	255.35 ± 28.42^#^
	5	6.21 ± 0.77	134.88 ± 6.85^†^	150.64 ± 14.05	232.66 ± 11.78^††##^
	10	6.58 ± 0.68^†^	101.37 ± 20.02^††^	131.01 ± 8.18^††^	206.05 ± 39.86^††##^
HFFD/FF	0.03	6.03 ± 1.02	100.82 ± 14.41^††^	120.65 ± 13.45^††^	176.68 ± 33.35^††##^

Mice were fed with normal diet (ND) or high fructose/fat diet (HFFD) for 3 months. Then, animals were fed with ND or HFFD without and with supplementation with the ethanol extract of Schisandrae Fructus (SF) seed (EtSF-S) or fenofibrate (FF). Thirty days later, serum and hepatic glucose was measured. In addition, the glucose levels of liver supernatants stored at 4°C or −20°C for 24 h were also determined for comparison. The concentrations of EtSF-S in chow diet were estimated on the basis of crude herbal material (SF seed). Values given are the mean ± SD, with *N* = 10. ^*∗*^*p* < 0.05, ^*∗∗*^*p* < 0.01 vs. ND alone; ^†^*p* < 0.05,^ ††^*p* < 0.01 vs. HFFD alone, ^##^*p* < 0.01 vs. the sample stared at −20°C.

**Table 4:** Effect of dietary supplementation with EtSF-S or FF on the hepatic, fat, and renal mass/index in ND- and HFFD-fed mice.

Groups	Dose (%, w/w)	A : liver		B : fat			C : kidney		
		Mass (g)	Index-1	Mass (g)	Index-1	Index-2	Mass (g)	Index-1	Index-2
ND-fed mice									
ND	-	1.26 ± 0.15	4.24 ± 0.36	0.50 ± 0.12	1.68 ± 0.42	1.76 ± 0.44	0.32 ± 0.04	1.09 ± 0.10	1.14 ± 0.10
ND/EtSF-S	10	1.36 ± 0.28	4.40 ± 0.65	0.32 ± 0.14 ^*∗∗*^	1.00 ± 0.46 ^*∗∗*^	1.09 ± 0.50 ^*∗∗*^	0.33 ± 0.05	1.07 ± 0.11	1.16 ± 0.12
ND/FF	0.03	1.82 ± 0.22 ^*∗∗*^	6.11 ± 0.60 ^*∗∗*^	0.28 ± 0.10 ^*∗∗*^	0.94 ± 0.34 ^*∗∗*^	1.00 ± 0.36 ^*∗∗*^	0.36 ± 0.03	1.19 ± 0.08 ^*∗*^	1.27 ± 0.09 ^*∗*^

HFFD-fed mice									
ND	**—**	1.53 ± 0.26	4.29 ± 0.38	0.48 ± 0.28	1.37 ± 0.66	1.47 ± 0.71	0.38 ± 0.05	1.06 ± 0.06	1.14 ± 0.06
HFFD	**—**	1.56 ± 0.18	4.25 ± 0.42	0.56 ± 0.26	1.63 ± 0.74	1.70 ± 0.77	0.36 ± 0.04	1.00 ± 0.08	1.04 ± 0.08
HFFD/EtSF-S	1	1.50 ± 0.19	4.25 ± 0.36	0.68 ± 0.31	1.98 ± 0.83	2.08 ± 0.87	0.35 ± 0.05	0.99 ± 0.13	1.04 ± 0.14
	5	1.79 ± 0.22^†^	5.13 ± 0.43^††^	0.58 ± 0.21	1.71 ± 0.60	1.80 ± 0.63	0.34 ± 0.04	0.97 ± 0.09	1.03 ± 0.10
	10	1.99 ± 0.14^††^	5.87 ± 0..32^††^	0.66 ± 0.14	1.97 ± 0.41	2.10 ± 0.44	0.32 ± 0.04^†^	0.94 ± 0.11	1.00 ± 0.12
HFFD/FF	0.03	2.02 ± 0.31^††^	5.46 ± 0.52^††^	0.63 ± 0.33	1.78 ± 0.86	1.93 ± 0.93	0.45 ± 0.07^††^	1.21 ± 0.14^†^	1.31 ± 0.15^†^

Mice were fed with normal diet (ND) or high fructose/fat diet (HFFD) for 3 months. Then, animals were fed with ND or HFFD without and with supplementation with the ethanol extract of Schisandrae Fructus (SF) seed (EtSF-S) or fenofibrate (FF). After 30 days of experiment, hepatic, fat (collected from uterine surface), or renal weight and index-1 (hepatic, fat, or renal weight/body weight × 100) and index-2 (fat or renal weight/body weight-hepatic weight × 100) were then measured. The concentrations of EStF-S in chow diet were estimated on the basis of crud herbal material (SF seed). Values given are the mean ± SD, with *N* = 10. ^*∗*^*p* < 0.05, ^*∗∗*^*p* < 0.01 vs. ND alone; ^†^*p* < 0.05, ^††^*p* < 0.01 vs. HFFD alone.

## Data Availability

The raw data supporting the conclusions of this article will be made available by the authors, without undue reservation, to any qualified researcher.

## References

[1] Lowndes J., Sinnett S., Yu Z., Rippe J. (2014). The effects of fructose-containing sugars on weight, body composition and cardiometabolic risk factors when consumed at up to the 90th percentile population consumption level for fructose. *Nutrients*.

[2] Schwab U., Lauritzen L., Tholstrup T. (2014). Effect of the amount and type of dietary fat on cardiometabolic risk factors and risk of developing type 2 diabetes, cardiovascular diseases, and cancer: a systematic review. *Food & Nutrition Research*.

[3] Ben M. D., Polimeni L., Baratta F., Pastori D., Loffredo L., Angelico F. (2014). Modern approach to the clinical management of non-alcoholic fatty liver disease. *World Journal of Gastroenterology*.

[4] Liu H., Lu H. Y. (2014). Nonalcoholic fatty liver disease and cardiovascular disease. *World Journal of Gastroenterology*.

[5] Liu Z. L., Xie L. Z., Zhu J., Li G. Q., Grant S. J., Liu J. P. (2013). Herbal medicines for fatty liver diseases. *Cochrane. Database Systematic Reviews*.

[6] Zelber-Sagi S., Lotan R., Shibolet O. (2013). Non-alcoholic fatty liver disease independently predicts prediabetes during a 7-year prospective follow-up. *Liver International*.

[7] Sham T. T., Chan C. O., Wang Y. H., Yang J. M., Mok D. K., Chan S. W. (2016). A review on the traditional Chinese medicinal herbs and formulae with hypolipidemic effect. *BioMed Research International*.

[8] Chun J. N., Cho M., So I., Jeon J.-H. (2014). The protective effects of Schisandra chinensis fruit extract and its lignans against cardiovascular disease: a review of the molecular mechanisms. *Fitoterapia*.

[9] Ye C., Han N., Teng F., Wang X., Xue R., Yin J. (2013). Extraction optimization of polysaccharides of Schisandrae Fructus and evaluation of their analgesic activity. *International Journal of Biological Macromolecules*.

[10] Lam P. Y., Ko K. M. (2012). Schisandrin B as a hormetic agent for preventing age-related neurodegenerative diseases. *Oxidative Medicine and Cellular Longevity*.

[11] Chen W.-W., He R.-R., Li Y.-F. (2011). Pharmacological studies on the anxiolytic effect of standardized Schisandra lignans extract on restraint-stressed mice. *Phytomedicine*.

[12] Li L., Zhang T., Zhou L. (2014). Schisandrin B attenuates acetaminophen-induced hepatic injury through heat-shock protein 27 and 70 in mice. *Journal of Gastroenterology and Hepatology*.

[13] Leong P. K., Chiu P. Y., Ko K. M. (2012). Prooxidant-induced glutathione antioxidant response in vitro and in vivo: a comparative study between schisandrin B and curcumin. *Biological and Pharmaceutical Bulletin*.

[14] Gao X. X., Meng X. J., Li J. H. (2008). Study on functions of active polysaccharide from Schisandra Chinensis (Turcz) Baill in reducing weight and fat. *Science Technology and Food Industry.*.

[15] Sun N., Pan S.-Y., Zhang Y. (2014). Dietary pulp from Fructus Schisandra Chinensis supplementation reduces serum/hepatic lipid and hepatic glucose levels in mice fed a normal or high cholesterol/bile salt diet. *Lipids in Health and Disease*.

[16] Wang X. Y., Yu Z. L., Pan S. Y. (2014). Supplementation with the extract of schisandrae fructus pulp, seed, or their combination influences the metabolism of lipids and glucose in mice fed with normal and hypercholesterolemic diet. *Evidence-Based Complementary and Alternative Medicine*.

[17] Pan S.-Y., Dong H., Zhao X.-Y. (2008). Schisandrin B fromSchisandra chinensisreduces hepatic lipid contents in hypercholesterolaemic mice. *Journal of Pharmacy and Pharmacology*.

[18] Pan S.-Y., Dong H., Yu Z.-L. (2007). Bicyclol, a synthetic dibenzocyclooctadiene derivative, decreases hepatic lipids but increases serum triglyceride level in normal and hypercholesterolaemic mice. *Journal of Pharmacy and Pharmacology*.

[19] Pan S.-Y., Yang R., Dong H., Yu Z.-L., Ko K.-M. (2006). Bifendate treatment attenuates hepatic steatosis in cholesterol/bile salt- and high-fat diet-induced hypercholesterolemia in mice. *European Journal of Pharmacology*.

[20] Zhu P. L., Pan S. Y., Zhou S. F. (2015). Effects of combined dietary supplementation with fenofibrate and Schisandrae Fructus pulp on lipid and glucose levels and liver function in normal and hypercholesterolemic mice. *Drug Design, Development and Therapy*.

[21] Crescenzo R., Bianco F., Coppola P. (2014). Fructose supplementation worsens the deleterious effects of short-term high-fat feeding on hepatic steatosis and lipid metabolism in adult rats. *Experimental Physiology*.

[22] Li M., Gu D., Xu N. (2014). Gut carbohydrate metabolism instead of fat metabolism regulated by gut microbes mediates high-fat diet-induced obesity. *Beneficial Microbes*.

[23] Shan B. X., Ai Z. F., Zeng S. F., Song Y. G., Zeng Q. (2020). Gut microbiome-derived lactate promotes to anxiety-like behaviors through GPR81 receptor-mediated lipid metabolism pathway. *Psychoneuroendocrinology*.

[24] Cani P. D. (2016). Changes in gut microbes and host metabolism: squaring the circle?. *Nature Reviews Gastroenterology & Hepatology*.

[25] Ference B. A., Yoo W., Alesh I. (2012). Effect of long-term exposure to lower low-density lipoprotein cholesterol beginning early in life on the risk of coronary heart disease. *Journal of the American College of Cardiology*.

[26] Anne L., Peters M. D. (2008). Clinical relevance of non-HDL cholesterol in patients with diabetes. *Clinical Diabetes*.

[27] Sniderman A., McQueen M., Contois J., Williams K., Furberg C. D. (2010). Why is non−high-density lipoprotein cholesterol a better marker of the risk of vascular disease than low-density lipoprotein cholesterol?. *Journal of Clinical Lipidology*.

[28] Yu X. L., Gao J., Li G. Q., Gao E. T. (2012). The relevance analysis between HDL/LDL ratio, LP (a), s-CRP and the degree of coronary artery disease. *Methods and Programs in Biomedicine*.

[29] Triolo M., Annema W., Dullaart R. P., Tietge U. J. (2013). Assessing the functional properties of high-density lipoproteins: an emerging concept in cardiovascular research. *Biomarkers in Medicine*.

[30] Kwon D. Y., Kim D. S., Yang H. J., Park S. (2011). The lignan-rich fractions of Fructus Schisandrae improve insulin sensitivity via the PPAR-*γ* pathways in in vitro and in vivo studies. *Journal of Ethnopharmacology*.

[31] Chu Z.-S., Yu Z.-L., Pan S.-Y. (2015). A comparative study between Wuweizi seed and its post-ethanol extraction residue in normal and hypercholesterolemic mice. *Lipids in Health and Disease*.

